# Results of a Pilot Study for a Pharmacy Discharge Review in Neurosurgical Patients: A Quality-Safety Initiative

**DOI:** 10.7759/cureus.36067

**Published:** 2023-03-13

**Authors:** Jessica C Greenwood, Keah Gutierrez, Michael McDermott

**Affiliations:** 1 Pharmacy/Neurology, Miami Neuroscience Institute, Miami, USA; 2 Pharmacy, Nova Southeastern University College of Pharmacy, Fort Lauderdale, USA; 3 Neurosurgery, Miami Neuroscience Institute, Miami, USA

**Keywords:** medication adherence, discharge counseling, pharmacist collaboration, neurosurgical, transition of care

## Abstract

Objective

A multidisciplinary collaboration between the neurosurgical team and the pharmacy was established to conduct a pilot study in which discharged neurosurgical patients from a community hospital would receive medication reconciliation services and counseling by a pharmacy specialist to determine the impact on patient safety, readmission rates, and medication compliance.

Methods

Pharmacists reviewed discharge medication reconciliations of neurosurgical patients to address any discrepancies with the nurse practitioners or physicians prior to discharge and provided discharge medication counseling to the patient/families at the bedside. The service was provided on weekdays during the eight-hour pharmacist shift in addition to other daily responsibilities. Data were analyzed by type and the total number of pharmacy interventions encountered during the discharge medication reconciliation process, time to complete services, and readmission rates. Lastly, the discharged neurosurgical patients that were not seen by pharmacists during the one-month pilot study were reviewed retrospectively to determine potential interventions.

Results

A total of 48 neurosurgical patients were discharged during the one-month pilot study; 27 patients received discharge medication reconciliation services and counseling from the pharmacy specialists. Sixty-three pharmacy interventions were accepted with prevention of medication errors/adverse drug reactions (21%, n=21) and addition of missing medication (21%, n=21) being the most common intervention types. The mean time to complete the services was 27 minutes and there was one non-medication-related readmission of the 27 patients seen. Twenty-one neurosurgical patients who were discharged without receiving services were reviewed retrospectively. It was determined that there was a potential for another 64 pharmacy interventions in which clarification of indication (33%, n=21) was the most common intervention type, followed by prevention of medication errors/adverse drug reactions (25%, n=16) and addition of missing medication (22%, n=14). There was a total of one medication-related readmission of the 21 patients not seen by the pharmacist during the pilot study.

Conclusion

The collaboration of pharmacists in the discharge process benefits neurosurgical patients by reducing the number of discrepancies when transitioning home and provides an additional layer of safety to reduce medication errors and/or prevent adverse events.

## Introduction

Neurosurgical patients are at risk for significant morbidity after discharge and require complex, costly perioperative care that extends into the outpatient setting [1-2]. Compared to other surgery subspecialties, neurosurgery carries a substantial risk of unplanned readmissions [3]. Complications of neurosurgical patients post-discharge include infections, intracranial hemorrhage, new sensory/motor deficits, wound complications, stroke, thromboembolic complications, and adverse drug reactions [3-7]. A majority of the unplanned readmissions are considered preventable and several patients have inconsistencies in their medical care [6-7]. This, in part, is due to the number of medications commonly prescribed to neurosurgical patients during inpatient hospital stays and continued after discharge. The medication lists for these patients are extensive, including opioids, anxiolytic therapies, seizure prophylaxis, blood pressure medications, antimicrobials, and anticoagulants, and warrant close monitoring and good compliance [8-9]. These medication classes have been found to account for a majority of medication discrepancies in the transition of care to an outpatient setting, a period during which patients are most vulnerable to readmissions [9-10]. In addition, several comorbidities have been identified as risk factors associated with unplanned readmissions such as hypertension, diabetes, chronic obstructive pulmonary disorder (COPD), coagulopathy, and chronic steroid use [3]. These findings suggest that quality improvement interventions should aim to optimize the medical management of comorbid diseases and to provide pre-discharge patient education and medication reconciliation for neurosurgical patients [3,6]. The objective of this pilot study is to assess the impact of a pharmacist-led discharge medication reconciliation and counseling service on neurosurgical patient safety and medication adherence.

## Materials and methods

This is a single-centered retrospective study conducted at a community hospital in Miami, FL, for patients admitted between April 12, 2021, and May 7, 2021. The hospital is an 850-bed community hospital, serving a large Hispanic population with a certified comprehensive stroke center and has a variety of neurosurgical sub-specialties. The study population was composed of adults aged 18 years or older presenting to the hospital and undergoing a neurosurgical intervention. Patients were excluded from the study if, after the neurosurgical intervention, they were transferred to a floor outside of the neurology unit for management of a co-morbidity. On weekdays, from 7 am to 3:30 pm during an eight-hour shift, the clinical pharmacists were informed of potential discharges via phone calls or huddles with neurosurgery. Once discharge medication reconciliations were completed by the discharging healthcare professional (advanced practice registered nurses (APRNs), neurosurgical APRNs, or hospitalists) the pharmacist would conduct a chart review and address any medication discrepancies with the discharging provider. Next, the pharmacist would counsel on medications, and provide the patient with a medication list that was generated from a software program entitled Med Action Pro. The pharmacy interventions would be documented on the electronic health records to specify the services provided and the discrepancies resolved. This was not a dedicated service line, as the clinical pharmacist had other daily staffing responsibilities. Retrospectively, all discharged neurosurgical patients were reviewed and the type and quantity of pharmacy interventions were documented.

## Results

**Figure 1:**
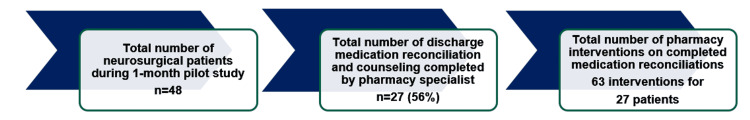
Total number of patients seen by pharmacists during discharge and the total number of pharmacy interventions during the one-month pilot study

From April 12, 2021, to May 7, 2021, there were 48 neurosurgical patients discharged from the neurosurgical unit. A total of 27 patients (56%) received discharge medication counseling and had their discharge medication list reviewed by a clinical pharmacist (Figure 1). The mean number of pharmacy interventions was 2.44 (range of 0-7 per patient). On average, the patients were admitted for a total of 5.08 days with a mode of 2 and a range of 1-16 days. The prescribers involved in the discharge medication reconciliation were as follows: 48% of the cases involved the hospitalist, 41% neurosurgical APRNs, and 11% were hospitalist APRNs. The mean time to complete a discharge medication reconciliation and counseling was 26.85 minutes, with a mode of 15 minutes, a median of 20 minutes, a range of 15-180 minutes, and an interquartile range of 5 minutes (Table 1). There were a total of two readmissions out of the 48 patients included in the pilot study. One readmission was medication related and occurred to a patient not seen by a pharmacist during the discharge process. The other readmission received pharmacy discharge services but was not medication related.

**Table 1:** Description of the length of stay, total number of pharmacy interventions per patient, time to complete discharge reconciliation/counseling, and review of prescribers involved in the discharge process for the 27 patients seen by pharmacy specialists during the one-month pilot study APRN: advanced practice registered nurse

Patients with Pharmacy Specialist Review (n=27)	
Length of Stay (days)	
Mean	5
Range	1-16
Mode	2
Pharmacy Interventions	
Total number of interventions	63
Mean interventions per patient	2
Range of interventions per patient	0-7
Total Time for Discharge Medication Reconciliation/Counseling (mins)	
Mean	27
Range	15-180
Mode	15
Number of Prescribers Involved in Medication Reconciliation	
Neurosurgery APRN	11 (41%)
Hospitalist	13 (48%)
Hospitalist APRN	3 (11%)

**Figure 2:**
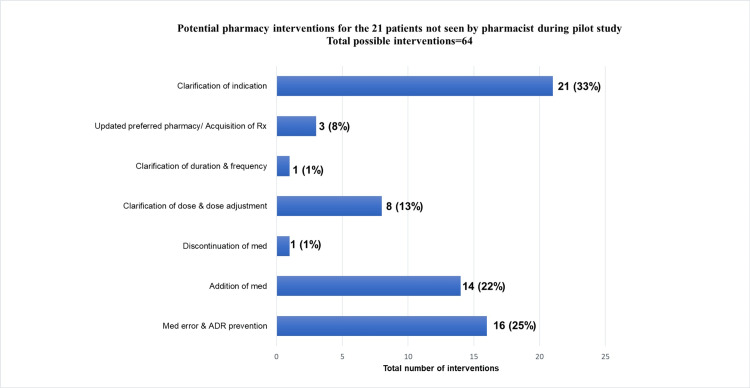
Classification of pharmacy interventions for the 27 patients seen by pharmacists during the one-month pilot study ADR: adverse drug reaction, Med: medication, Rx: prescription

A total of 63 pharmacy interventions were documented during the one-month pilot study for the 27 patients who received discharge services from the pharmacy (Figure 1). The interventions were classified as the following: 21% (n=13) medication error/adverse drug reaction prevention, 21% (n=13) addition of medication, 16% (n=10) discontinuation of medication, 13% (n=8) clarification of dose and dose adjustments, 10% (n=6) clarification of duration & frequency, 10% (n=6) therapeutic duplication, 8% (n=5) updated preferred pharmacy and acquisition of prescription, and 3% (n=2) clarification of indication (Figure 2).

**Figure 3:**
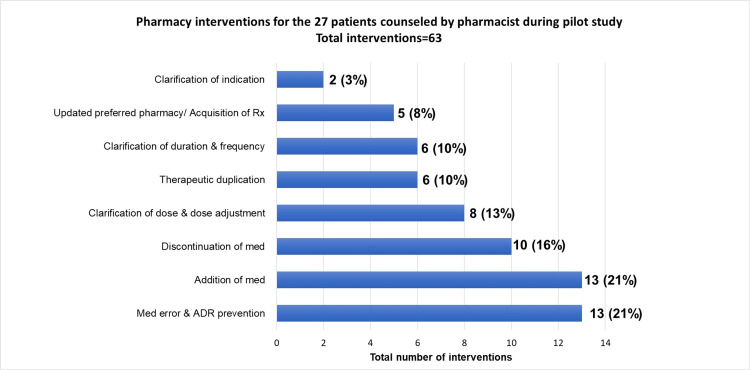
Classification of the potential pharmacy interventions for the 21 patients not seen by the pharmacist during the one-month pilot study ADR: adverse drug reaction, Med: medication, Rx: prescription

There were 21 patients that were missed during the one-month pilot study and were not seen by the clinical pharmacist prior to discharge. These patient charts were reviewed for potential interventions. It was determined that there was a possibility for an additional 64 pharmacy interventions, which would have been classified as follows: 33% (n=21) clarification of indication, 25% (n=16) medication error/adverse drug reaction prevention, 22% (n=14) addition of medication, 13% (n=8) clarification of dose and dose adjustments, 5% (n=3) updated preferred pharmacy and acquisition of prescription, 1% (n=1) discontinuation of medication, and 1% (n=1) clarification of duration and frequency (Figure 3). 

## Discussion

Pharmacists' positive impact on the critically ill population has been well-established. They are recognized as essential members of the critical care multidisciplinary teams by several pharmacy and interprofessional organizations [11-15]. A position paper released by the Society of Critical Care Medicine, American College of Clinical Pharmacy Critical Care Practice and Research Network, and American Society of Health-Systems Pharmacists defined the scope of pharmacy services within the intensive care unit (ICU) while an article released by the Neurocritical Care Society designates pharmacists as “essential” to the neurocritical care team [14-15]. The articles highlighted that the role of the critical care pharmacist commenced from verification of the medication history to inpatient medication management to the reconciliation of the discharge medication regimen. Studies have shown the effectiveness of pharmacist-led medication reconciliation in improving post-discharge medication safety and transitions of care [11,16-19]. However, there is still a paucity of data regarding the benefits of discharge services of pharmacists specifically for the neurosurgical patient population, with only two published studies to date [11,19].

This pilot study provides further evidence that the involvement of a pharmacy specialist in the discharge medication reconciliation process can avoid medication errors, improve patient/family understanding of medication administration, and thus potentially improve patient safety/outcomes following discharge. Discrepancies ranged from 0 to 7, with the mean number of interventions per patient being 2.44. In general, patients with polypharmacy and extensive medication history required more time and have greater potential for discrepancies.

The most common pharmacy intervention type was the prevention of medication errors or adverse drug reactions. Mostly, this included the holding and restarting of antithrombotic and oral oncolytic medications and ensuring patients had clear instructions or a calendar specifying dates of hold parameters. In addition, many of the neurosurgical patients had prescriptions for pain medication prior to admission, which was modified or discontinued at discharge. These services from the pharmacist informed the patient of home prescriptions that needed to be stopped or set aside to prevent a medication error or opioid duplication. Another common intervention was the addition of a medication, which was often reflected by the addition of “over-the-counter” pain medications for multimodal pain management, and bowel regimens to prevent constipation from opioid use. The discontinuation of medication was the third most common intervention, which included the removal of medications that no longer were indicated. At times, patients' doses were modified based on laboratory values, blood pressure, or heart rate; renal dose adjustments, and changes in blood pressure medications were the most frequent modifications. Furthermore, the most common clarifications for medications were regarding the duration of therapy or administration frequency. For example, antiepileptic medications ordered inpatient in the short term (i.e. 5-7 days postoperatively) for prophylaxis were prescribed for one month upon discharge. In addition, custom steroid taper schedules and calendars were generated for neurosurgical patients to prevent confusion about duration and doses. A few of the discrepancies involved therapeutic duplication which was seen due to formulary interchanges. In some cases, the patient's preferred pharmacy was updated, and prescriptions needed to be resent. Lastly, the pharmacist had to clarify the indication of medications; the parameters for “PRNs” or as-needed pain medications were the most frequent intervention to ensure that the patient had clear instructions on how many tablets or which medication to use for all pain scales from 1 to 10.

A limitation of the study was that discharge medication reconciliation/counseling was not a dedicated service from the pharmacist and was limited to weekdays, therefore, patients discharged during the evening or weekends were not seen. In addition, there is no alert system in the electronic health record to identify patients ready for discharge. This has prompted our team to work with information technology services to create such an alert for the pharmacy team. Another limitation was the turnover of the hospitalist team, which alternated every week as this led to physicians/APRNs who were not accustomed to collaborating with the pharmacist during the discharge medication reconciliation process. All these limitations contributed to missed opportunities as seen by the results discussed in Figure 3.

## Conclusions

Neurosurgical patients are predisposed to many post-discharge medication errors and post-surgical complications and an important part of their care includes compliance with medication orders. This study demonstrated that pharmacist collaboration in the discharge process for neurosurgical patients allowed for the prevention of medication errors and adverse drug reactions for 21% of patients included in the pilot, and 25% of those outside of the pilot study and reviewed retrospectively. This included the holding/restarting of antithrombotic therapy, communicating discontinuation/changes to home medications, securing prescriptions from retail pharmacies, and optimizing pain management/ steroid tapers. Patients that benefit the most from pharmacist discharge medication reconciliation and counseling are patients with polypharmacy or if the patient is continuing high-risk medications (i.e. antithrombotics, opioids, anxiolytics, antiepileptics, etc.). Lastly, the addition of pharmacists in the transition of care of neurosurgical patients can lead to improved medication adherence and may lead to increased patient satisfaction and/or prevent medication-related readmissions.
